# Supplementary material for: COVID-19 Stress and Teachers Well-Being: The Mediating Role of Sense of Coherence and Resilience
Source: Eur J Investig Health Psychol Educ. 2022 Dec 21;13(1):1–22. doi: 10.3390/ejihpe13010001 (PMC9858340; doi:10.3390/ejihpe13010001)
Supplement: Supplementary file 1 [file ejihpe-13-00001-s001.zip › ejihpe-1942556-supplementary.pdf]

Supplementary Files

Supplementary S1-Perceived Stress Scale of COVID-19 (PSS-10-C)

**Items**	**Original**	**Amharic**
	During the last 7 days	ባለፉት 7 ቀናት ውስጥ
Item 1	I have had the feeling that the pandemic's spread will cause something significant to occur without warning.	ከወረርሽኙ ጋር ባልታሰበ ሁኔታ አንድ ከባድ ነገር እንደሚከሰት ይሰማኛል::
Item 2	I have felt that the pandemic has made difficult for me to control over the crucial aspects of my life.	በወረርሽኙ ምክንያት በሕይወቴ ውስጥ አስፈላጊ ነገሮችን መቆጣጠር እንደማልችል ይሰማኛል::
Item 3	I have experienced anxiety and worry due to the pandemic	ስለ ወረርሽኙ ፍርሃት ወይም ጭንቀት ይሰማኛል::
Item 4	I have felt confidence in my capacity to manage any personal issues caused by the epidemic.	ከወረርሽኙ ጋር ተያይዞ የሚከሰቱትን የግል ችግሮቼን የመቋቋም ችሎታ እንዳለኝ እርግጠኛ ነኝ ፡፡
Item 5	I have felt optimistic that things are going well with the epidemic	በወረርሽኙም: ውስጥ ሆነን ነገሮች በጥሩ ሁኔታ እንደሚሄዱ ብሩህ ተስፋ ይሰማኛል ፡፡
Item 6	I have felt unable to cope with the things I have to do to monitor for a possible infection.	ሊመጣ ከሚችል ኢንፌክሽን ለመከላከል ማድረግ ያለብኝን ነገሮች መቋቋም እንደማልችል ይሰማኛል::
Item 7	I have felt that I can control the difficulties that could appear in my life as a result of the infection.	በኢንፌክሽን ምክንያት በሕይወቴ ውስጥ ሊታዩ የሚችሉትን አስቸጋሪ ችግሮችን መቆጣጠር እንደምችል ይሰማኛል::
Item 8	I have felt in control of the pandemic in everyway.	ከወረርሽኙ ጋር በተያያዘ በሁሉም ነገር በቁጥጥር ስር እንደሆንኩ ይሰማኛል::
Item 9	I have been upset that things related to the epidemic are out of my control.	ከወረርሽኙ ጋር የተያያዙ ነገሮች ከቁጥጥሬ ውጭ በመሆናቸው ተበሳጭቻለሁ ፡፡

Item 10	I have felt that the difficulties are increasing in these days of the epidemic and I feel unable to overcome them of.	በእነዚህ የወረርሽኙ ቀናት ውስጥ ችግሮቹ እየጨመሩ እንደመጡ እና እነዚህን ለማሸነፍ እንደማልችል ይሰማኛል ።

Scoring

No.	Original-English					Amharic Version				
	0= Never	1= almost never	2= occasionally	3= almost always	4= Always	0=በጭራሽ	1=በጭራሽ ማለት ይቻላል	2=አልፎ አልፎ	3=ሁልጊዜ ማለት ይቻላል	4=ሁልጊዜ
1	0	1	2	3	4	0	1	2	3	4
2	0	1	2	3	4	0	1	2	3	4
3	0	1	2	3	4	0	1	2	3	4
4	4	3	2	1	0	4	3	2	1	0
5	4	3	2	1	0	4	3	2	1	0
6	0	1	2	3	4	0	1	2	3	4
7	4	3	2	1	0	4	3	2	1	0
8	4	3	2	1	0	4	3	2	1	0
9	0	1	2	3	4	0	1	2	3	4
10	0	1	2	3	4	0	1	2	3	4

Note: Items scored directly from **0 to 4 were 1, 2, 3, 6, 9, and 10**; reversed scores were items **4, 5, 7, and 8 from** 4 to 0 (Campo-Arias et al., 2020).

Supplementary S2. The Brief Resilience Scale

	Original English Version	Amharic Version

1	I tend to bounce back quickly after hard times	ከአስጨናቂ ጊዜ በኋላ በፍጥነት ወደ ነበርኩበት የመመለስ አዝማሚያ አለኝ
2	I have a hard time making it through stressful events (R)	በአስጨናቂ ክስተቶች ጊዜ ነገሮችን ለመቋቋም በጣም ይቸግረኛል
3	It does not take me long to recover from a stressful event	ከአስጨናቂ ክስተት ለማገገም ብዙ ጊዜ አይወስድብኝም
4	4. It is hard for me to snap back when something bad happens (R)	መጥፎ ነገር በሚከሰት ጊዜ ቶሎ ለመመለስ ለእኔ ከባድ ነው
5	I usually come through difficult times with little trouble	ብዙውን ጊዜ በአስቸጋሪ ጊዜያት ውስጥ በትንሽ ችግር እወጣለሁ
6	I tend to take a long time to get over set-backs in my life (R)	በሕይወቴ ውስጥ ወደ ትክክለኛ ህይዎቴ ለመመለስ ብዙ ጊዜ ይወስድብኛል

	**Scoring**			
	Original-English		Amharic Version	
	1	5	1	5
To all items (1-7)	Strongly Disagree	Strongly Agree	በጣም አልስማማም	በጣም እስማማለሁ

Supplementary S3. Sense of Coherence-13 Scale

No.	original	Amharic

1	Do you have the feeling that you don’t really care about what goes on around you?	በአካባቢዎ ለሚከናዎኑ ነገሮች ግድ የለኝም የሚል ስሜት አሎት?
2	Have you ever been surprised by the behavior of people you thought you knew well?	በደንብ አውቃቸዋለሁ ብለዉ ያሰቧቸው ሰዎች ባህሪ አስገርሞዎት ያውቃል?
3	Has it happened that people whom you counted on disappointed you?	እርስዎ ያመኗቸው ሰዎች ተስፋ አስቆርጦዎታል?
4	Until now your life has had	እስካሁን በህይዎት እኖራለሁ
5	Do you have the feeling that you’re being treated unfairly?	ኢ-ፍትሃዊ በሆነ መንገድ እንደሚዎስድዎት ይሰማዎታል
6	Do you have the feeling that you are in an unfamiliar situation and don’t know what to do?	በማይታወቅ ሁኔታ እንደሆኑ እና ምን ማድረግ እንዳለበዎ የማያውቁበት ስሜት አለዎት?
7	Doing the thing you do every day is	በየቀኑ የሚያደርጉትን ነገር ማድርግ?
8	Do you have very mixed-up feelings and ideas?	በጣም የተደበላለቀ ስሜቶች እና ሃሳቦች አሉዎት?
9	Does it happen that you have feelings inside you would rather not feel	በውስጥዎ ስሜቶች ቢኖርዎትም የማይሰማዎት ነገር ይከሰታል?
10	Many people even those with a strong character – sometimes feel like sad sacks (losers) in certain situations. How often have you felt this way in the past?	ብዙ ሰዎች ጥሩ ፀባይ ያሏቸው እንኳን አንዳንድ ጊዜ በተወሰኑ ሁኔታዎች ውስጥ የባዶነት ና የሃዘን(የተሸናፊነት) ይሰማቸዋል።ከዚህ በፊት ምን ያህል ጊዜ እነደዚህ አይነት ስሜት ተሰምትዎት ያውቃል?
11	When something happened, have you generally found that?	የሆነ ነገር ሲከሰትበአጠቃላይ ያንን ነገር ምን ያክል አግኝተዋል?
12	How often do you have the feeling that there’s little meaning in the things you do in your daily life?	በእለት ተዕለት ህይዎትዎ ውስጥ የሚያከናውኗቸው ነገሮች ትንሽ ትርጉም እንዳለው ምን ያህል ጊዜ ይሰማዎታል?
13	How often do you have feelings that you’re not sure you can keep under control?	በቁጥጥር ስር ማዋል እንደምትችል እርግጠኛ ያልሆንክባቸው ስሜቶች ስንት ጊዜ አለዎት?

Scoring

	**Original-English**		**Amharic Version**	
	1	7	1	7
1	Seldom or never	Very often	አልፎ አልፎ ወይም በጭራሽ	በጣም ብዙ ጊዜ
2	Never happened	Always happened	በጭራሽ አልተከሰተም	ሁል ጊዜም ተከሰተ
3	Never happened	Always happened	በጭራሽ አልተከሰተም	ሁል ጊዜም ተከሰተ
4	No clear goals or purposes at all	Very clear goals or purposes	በጭራሽ ግልጽ ግቦች ወይም ዓላማዎች የሉም	በጣም ግልጽ የሆኑ ግቦች ወይም ዓላማዎች
5	Very often	Seldom or never	በጣም ብዙ ጊዜ	አልፎ አልፎ ወይም በጭራሽ
6	Very often	Seldom or never	በጣም ብዙ ጊዜ	አልፎ አልፎ ወይም በጭራሽ
7	A source of deep pleasure and satisfaction	source of pain and boredom	ጥልቅ ደስታ እና እርካታ ምንጭ	የህመምና መሰላቸት ምንጭ
8	Very often	Seldom or never	በጣም ብዙ ጊዜ	አልፎ አልፎ ወይም በጭራሽ
9	Very often	Seldom or never	በጣም ብዙ ጊዜ	አልፎ አልፎ ወይም በጭራሽ
10	Very often	Seldom or never	በጣም ብዙ ጊዜ	አልፎ አልፎ ወይም በጭራሽ
11	Over- or underestimate it's importance	saw things in the right proportion	ከመጠን በላይ - ወይም አቅልሎ ማየት አስፈላጊ ነው	ነገሮችን በትክክለኛው መጠን ተመልክቷል
12	Very often	Seldom or never	በጣም ብዙ ጊዜ	አልፎ አልፎ ወይም በጭራሽ
13	Very often	Seldom or never	በጣም ብዙ ጊዜ	አልፎ አልፎ ወይም በጭራሽ

Supplementary S4. Teacher Well-Being (የአውንታዊ ስነ -ልቦና ለኬት)

Block		Original English Version	Amharic Version
Block 1	A1	How much of the time do you feel you are making progress towards accomplishing your goals?	ግቦችን ለማሳካት ምን ያህል የጊዜ ሂደት እንደወሰደበዎት ይሰማዎታል?
	E1	How often do you become absorbed in what you are doing?	ምን እየሰሩ ነዉ፤ምን ያህል ጊዜ ሙሉ በሙሉ በስራ ተጠምደዋል?
	P1	In general, how often do you feel joyful?	በአጠቃላይ ፤ምን ያህል ጊዜ ደስታ ይሰማዎታል?
	A2	How often do you achieve the important goals you have set for your- self?	ለራስዎ ያስቀመጧቸዉን በጠማ አስፈላጊ ግቦች በምንያክል ጊዜ ያሳካሉ?
Block 2	M1	In general, to what extent do you lead a purposeful and meaningful life?	በአጠቃላይ ዓላማ እና ትርጉም ያለዉ ህይዎት የሚመሩት እስከምን ድረስ ነዉ?
	R1	To what extent do you receive help and support from others when you need it?	በሚፈልጉት ጊዜ ከሌሎች ምን ያህል ድጋፍ ና እገዛ ያገኛሉ?
	M2	In general, to what extent do you feel that what you do in your life is valuable and worthwhile?	በአጠቃላይ በሕይዎትዎ ውስጥ የሚያደርጉት ነገርትርጉም ያለው ?እንደሆነ እስከ ምን ድረስ ይሰማዎታል
	E2	In general, to what extent do you feel excited and interested in things?	በአጠቃላይ እስከ ምን ድረስ ደስታ ና የነገሮች ፍላጎት እንዳለዎት ይሰማዎታል
Block 3	P2	In general, how often do you feel positive?	በአጠቃላይ ምን ያህል ጊዜ አውንታዊ ስሜት ይሰማዎታል
	A3	How often are you able to handle your responsibilities?	ምን ያህል ጊዜኃላፊነቶችን መወጣትይችላሉ
	E3	How often do you lose track of time while doing something you enjoy?	የሚወዱትን ነገር ሲያደርጉ ምን ያህል ጊዜዎን ያጠፋሉ
Block 4	R2	To what extent do you feel loved?	ምን ያህል እንደተወደዱ ይሰማዎታል?
	M3	To what extent do you generally feel you have a sense of direction in your life?	በአጠቃላይ ምን ያህል ሕይዎትዎን የመምራት ስሜት እንዳለዎት ይሰማዋታል?
	R3	How satisfied are you with your personal relationships?	በግል ግንኙነትዎ ምን ያህል እርካታ ይሰማዎታል?

	P3	In general, to what extent do you feel contented?	በአጠቃላይ ምን ያህል እርካታ ይሰማዎታል?	
	Original-English		Amharic Version	
Block	0	10	0	10
1	Never	always	በጭራሽ	ሁልጊዜ
2	not at all	completely	በጭራሽ	ሙሉ በሙሉ
3	Never	always	በጭራሽ	ሁልጊዜ
4	not at all	completely	በጭራሽ	ሙሉ በሙሉ
